# Potential pharmacokinetic interactions with concurrent use of herbal medicines and a ritonavir-boosted COVID-19 protease inhibitor in low and middle-income countries

**DOI:** 10.3389/fphar.2023.1210579

**Published:** 2023-07-12

**Authors:** Dallas J. Smith, Huichang Bi, Josias Hamman, Xiaochao Ma, Constance Mitchell, Kumbukani Nyirenda, Tsitsi Monera-Penduka, Hellen Oketch-Rabah, Mary F. Paine, Syril Pettit, Wihan Pheiffer, Richard B. Van Breemen, Michelle Embry

**Affiliations:** ^1^ Epidemic Intelligence Service, Centers for Disease Control and Prevention, Atlanta, GA, United States; ^2^ COVID-19 Response International Task Force, Centers for Disease Control and Prevention, Atlanta, GA, United States; ^3^ School of Pharmaceutical Sciences, Southern Medical University, Guangzhou, China; ^4^ Centre of Excellence for Pharmaceutical Sciences (Pharmacen™), Faculty of Health Sciences, North-West University, Potchefstroom, South Africa; ^5^ Department of Pharmaceutical Sciences, School of Pharmacy, University of Pittsburgh, Pittsburgh, PA, United States; ^6^ Health and Environmental Sciences Institute, Washington, DC, United States; ^7^ Department of Pharmacy, Kamuzu University of Health Sciences, Blantyre, Malawi; ^8^ Research Unit for Safety of Herbs and Drugs, University of Zimbabwe, Harare, Zimbabwe; ^9^ United States Pharmacopeia, Rockville, MD, United States; ^10^ Department of Pharmaceutical Sciences, College of Pharmacy and Pharmaceutical Sciences, Washington State University, Spokane, WA, United States; ^11^ DSI/NWU Preclinical Drug Development Platform, Faculty of Health Sciences, North-West University, Potchefstroom, South Africa; ^12^ Department of Pharmaceutical Sciences, College of Pharmacy, Oregon State University, Corvallis, OR, United States

**Keywords:** COVID-19, herb-drug interaction (HDI), drug-drug interaction (DDI), PAXLOVID, herbal medicine

## Abstract

The COVID-19 pandemic sparked the development of novel anti-viral drugs that have shown to be effective in reducing both fatality and hospitalization rates in patients with elevated risk for COVID-19 related morbidity or mortality. Currently, nirmatrelvir/ritonavir (Paxlovid™) fixed-dose combination is recommended by the World Health Organization for treatment of COVID-19. The ritonavir component is an inhibitor of cytochrome P450 (CYP) 3A, which is used in this combination to achieve needed therapeutic concentrations of nirmatrelvir. Because of the critical pharmacokinetic effect of this mechanism of action for Paxlovid™, co-administration with needed medications that inhibit or induce CYP3A is contraindicated, reflecting concern for interactions with the potential to alter the efficacy or safety of co-administered drugs that are also metabolized by CYP3A. Some herbal medicines are known to interact with drug metabolizing enzymes and transporters, including but not limited to inhibition or induction of CYP3A and P-glycoprotein. As access to these COVID-19 medications has increased in low- and middle-income countries (LMICs), understanding the potential for herb-drug interactions within these regions is important. Many studies have evaluated the utility of herbal medicines for COVID-19 treatments, yet information on potential herb-drug interactions involving Paxlovid™, specifically with herbal medicines commonly used in LMICs, is lacking. This review presents data on regionally-relevant herbal medicine use (particularly those promoted as treatments for COVID-19) and mechanism of action data on herbal medicines to highlight the potential for herbal medicine interaction Herb-drug interaction mediated by ritonavir-boosted antiviral protease inhibitors This work highlights potential areas for future experimental studies and data collection, identifies herbal medicines for inclusion in future listings of regionally diverse potential HDIs and underscores areas for LMIC-focused provider-patient communication. This overview is presented to support governments and health protection entities as they prepare for an increase of availability and use of Paxlovid™.

## Introduction

The development of novel anti-viral drugs represents a critical step forward in the global control of severe acute respiratory syndrome coronavirus-2 (SARS-CoV-2), also known as COVID-19. Access to these medications for use by patients at elevated risk for COVID-19-related morbidity or mortality has dramatically lowered hospitalization and fatality rates, and reduced the overall burden of illness as well (Petty and Malani, 2022). Of these novel drug products, nirmatrelvir/ritonavir (Paxlovid™) fixed-dose combination is recommended by the World Health Organization for treatment of COVID-19 (WHO, 2022). Although access to this and other COVID-19 treatments is limited (Usher, 2022), progress has been made to increase access in 95 low- and middle-income countries (LMICs) via a partnership between the manufacturer and the Medicines Patent Pool (Pfizer, 2021).

**FIGURE 1:**
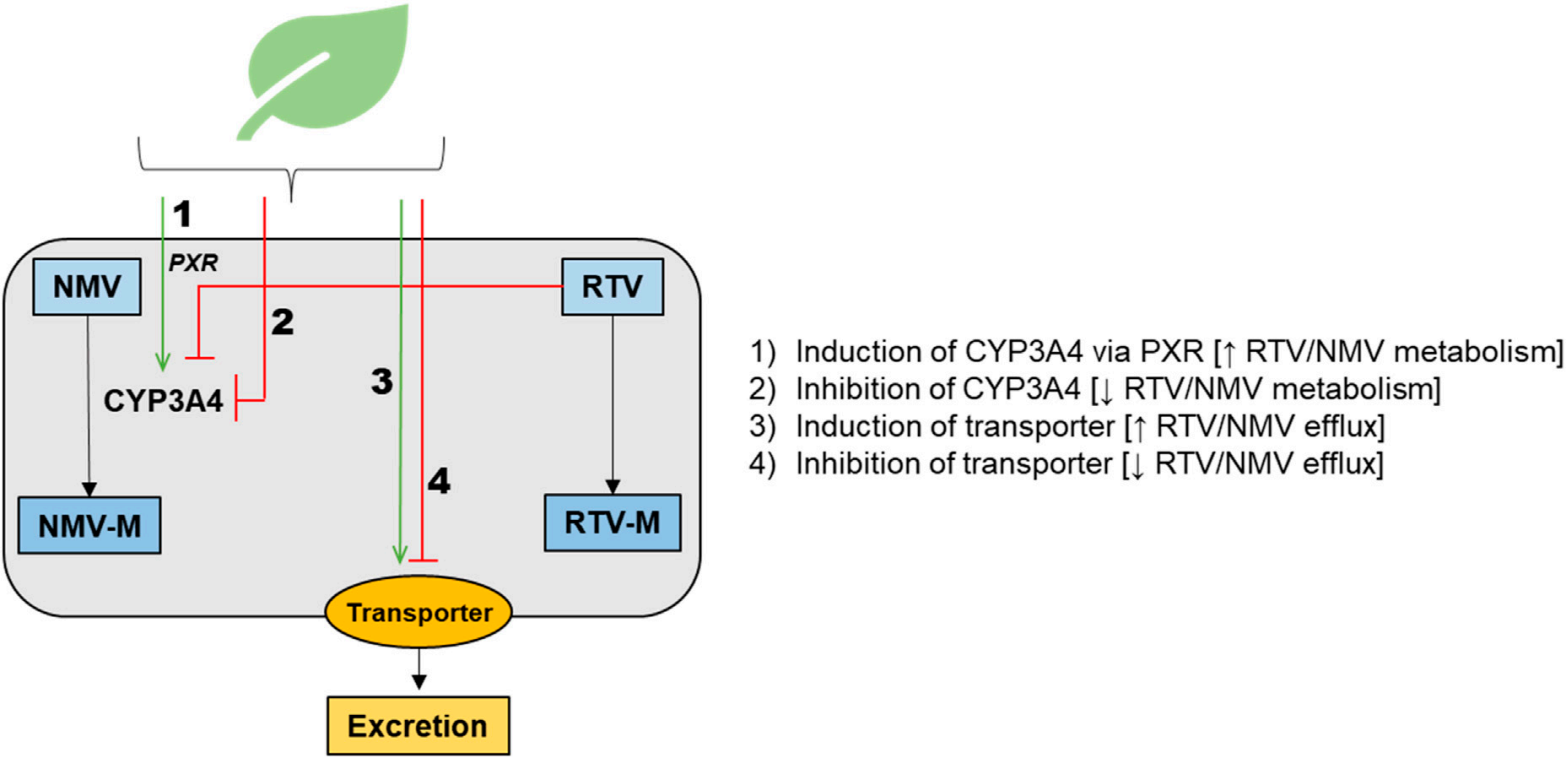
Schematic representation of the main potential mechanisms for herb-drug interactions involving Paxlovid™, highlighting pathways that could impact systemic and/or tissue concentrations of nirmatrelvir (NMV), ritonavir (RTV), NMV metabolite(s) (NMV-M), and/or RTV metabolite(s) (RTV-M). PXR, pregnane X receptor; CYP3A4, cytochrome P450 3A4.

Ritonavir, a protease inhibitor with antiviral properties, has been used for decades as part of HIV Highly Active Antiretroviral Therapy (HAART). Ritonavir acts as a pharmacokinetic boosting agent for other antiviral agents contained in both HAART and Paxlovid™ by inhibiting the prominent drug-metabolizing enzyme cytochrome P450 (CYP) 3A, facilitating the achievement of therapeutic concentrations by other co-administered antiviral drugs metabolized by CYP3A (Croxtall and Perry, 2010). Because of this mechanism of action for Paxlovid™, the U.S. Food and Drug Administration (FDA) and European Medicines Agency (EMA) labelling states that co-administration with certain medications that inhibit or induce CYP3A is contraindicated. This designation reflects concern for potential drug-drug interactions (DDIs) that may alter the efficacy of ritonavir or nirmatrelvir or alter the efficacy or safety of co-administered drugs metabolized by CYP3A (Marzolini et al., 2022). In addition to pharmaceutical medications, some herbal medicines (Gurley, 2012; Sprouse and van Breemen, 2016) are known to interact with drug metabolizing enzymes and transporters, including but not limited to inhibition or induction of CYP3A and the apically located efflux transporter P-glycoprotein (P-gp). As of the writing of this manuscript, only one herbal medicine, St. John’s Wort (*Hypericum perforatum*), is included in the list of contraindicated substances highlighted in the FDA or EMA fact sheets for healthcare providers (USFDA, 2022a; Paxlovid, 2022). As Paxlovid™ becomes more globally available—particularly throughout the African and Asian continents—it will be critical to contextualize it within a background of concomitant herbal medicine use in those regions. Such use is both substantially greater and distinct from that in North America and Europe. Use of herbal medicines and supplements during the COVID-19 pandemic increased significantly, with several countries actively promoting herbal treatments combined with pharmaceuticals (Ang et al., 2020) and prompting the Africa Centers for Disease Control and Prevention to issue a warning statement about herbal medicines as treatments for COVID-19 (Africa Centers for Disease Control and Prevention, 2020). Although many studies have evaluated the utility of herbal medicines for COVID-19 treatments, particularly in LMICs (Dwarka et al., 2020; Adeleye et al., 2021; Attah et al., 2021; Beressa et al., 2021; Gajewski et al., 2021; Mphekgwana et al., 2021; Akindele et al., 2022), well characterized and readily accessible information on potential herb-drug interactions (HDIs) involving Paxlovid™ is lacking (Bertuccioli et al., 2022). Conceptually Paxlovid™ interaction with herbs and other drugs may involve the pathways in Figure 1 and could impact systemic and/or tissue concentrations of nirmatrelvir (NMV), ritonavir (RTV), NMV metabolite(s) (NMV-M), and/or RTV metabolite(s) (RTV-M). PXR, pregnane X receptor; CYP3A4, cytochrome P450 3A4.

To the authors’ knowledge, this review is the first to present data on regionally relevant herbal medicine use (particularly those promoted as treatments for COVID-19) in LMICs and mechanistic data on herbal medicines to discuss the potential for ritonavir-boosted antiviral protease inhibitor-mediated HDIs. This work highlights potential areas for future experimental studies and data collection, identifies herbal medicines for inclusion in future listings of regionally diverse potential HDIs, and suggests areas for LMIC-focused provider-patient communication. This summary aims to support governments and health protection entities as they prepare for the global availability and use of Paxlovid™.

Because the current Emergency Use Authorizations in the United States for Paxlovid™ are focused on CYP3A-mediated interactions (USFDA M., 2022; Paxlovid, 2022), we direct our efforts on this mechanism, as most available information for herbal medicines is related to CYP3A (or CYPs more broadly). Other mechanisms underlying potential HDIs (e.g., inhibition or induction of P-gp and other transporters) are recognized, and additional research is needed to further explore these pathways. This review highlights the potential for herbal medicines to alter the efficacy or safety of nirmatrelvir/ritonavir. The reverse situation, wherein nirmatrelvir/ritonavir could alter the efficacy or safety of the herbal medicine, is also possible but is beyond the scope of this review.

This work is not a systematic review of potential novel HDIs with COVID-19 treatments but rather presents data derived from a limited pool of clinical and nonclinical studies for herbal medicines identified as commonly used in parts of Africa or Asia, or those used or proposed for use in the treatment of COVID-19 in these regions. This review draws on data from the peer-reviewed literature, as well as expert opinion from pharmacognosists, pharmacologists, toxicologists, researchers—including in-country experts in these areas—to inform initial case studies on potential HDIs involving herbal medicines and Paxlovid™ in low and middle-income regions in Africa and Asia. The description of plants as treatments in this review are not meant to support the reported efficacy of the plants, but to describe their use and discuss potential interactions (Collins et al., 2020; Mitchell et al., 2022). Herbal medicines and preparations derived from them (e.g., extracts or tinctures) are complex mixtures, containing hundreds to thousands of individual chemicals (or phytochemicals). This natural complexity (and variability) makes it difficult to assess the efficacy, safety, or herbal-drug interactions of any one preparation without sufficient chemical characterization (Collins et al., 2020; Mitchell et al., 2022). However, we hope this review can stimulate further research and potentially alert those taking herbal medicines and anti-virals to treat COVID-19 about potential interactions with Paxlovid.

## Methods

The author team identified a list of herbal medicines and plants for potential inclusion in this article based upon the following criteria:

• Expert opinion of author team based on local practices and availability in their county or region.

• Initial data of common use in LMICs in Africa and Asia.

• Initial data of promotion or use specifically as a treatment for COVID-19.

• Potential for pharmacokinetic interactions with Paxlovid™ based on available mechanistic or clinical evidence (e.g., modelling of known phytochemicals and *in vitro*, *in vivo*, or clinical data).

The herbal medicines/plants on this list were then further characterized via a non-systematic literature search to identify HDIs associated with the plant, mechanistic studies on the plant, and adverse event reports associated with the plant’s use. The non-systematic literature review informs on HDIs rather than give an all-compassing review of each botanical. The search was conducted in English using the following sources: PubMed, Web of Science, Google Scholar, Science Direct, China National Knowledge Infrastructure, and the online ethnobotanical database. This activity was reviewed by CDC and was conducted consistent with applicable federal law and CDC policy.^1^

## Results

A summary of the plants examined in this review is presented in Table 1.

**TABLE 1:** Summary of Herbal Medicines highlighted in this review.

Scientific name	Common Name(s)	Native region(s)	Regions/countries commonly used	Plant parts(s)/Preparation	Common traditional (non-COVID-19) therapeutic use(s)/Biological activities
*Andrographis paniculate*	Green chiretta	India Sri Lanka	Southeast Asia (Thailand), China	Leaf extract	• Stomachache
					• Inflammation
					• Pyrexia
					• Fevers
					• Loss of appetite
					• Irregular stools
					• Diarrhoea
*Artemisia annua*	Sweet wormwood	Asia	Asia; Africa	Dried leaves; tea	• Malaria
					• Inflammation (e.g., osteoarthritis)
					• Viral infections
					• Bacterial infections
					• Fevers
					• Cancer
*Bowiea volubilis*	Climbing onion	Southern Africa	Southern Africa	Decoction of crushed fresh bulbs & water	• Dermatological
					• Sore eyes
					• Urinary complications
					• Infertility
					• Abortion
*Curcuma longa*	Turmeric	South Asia	Widespread	Dried rhizomes	• Ulcers
					• Allergy
					• Pain
					• Inflammation
					• Liver disorders
*Glycyrrhizae radix et rhioma*	Licorice	West Asia, North Africa, S. Europe	China	Dried root; dried rhizome	• Allergies
					• Inflammation
					• Viral infections
					• Cancer
*Harpagophytum procumbens*	Devil’s claw	Southern Africa	Southern Africa	Dried root/tuber extract	• Rheumatoid arthritis
					• Osteo arthritis
					• Pain
					• Kidney inflammation
					• Dyspepsia
					• Fever
					• Wound healing
*Hypoxis haemercollidea*	African potato	Southern Africa	Southern Africa	Decoction of corms	• HIV/AIDS
					• Tuberculosis
					• Cancer
					• Headache
					• Dizziness
					• Ulcers
					• Seizures
					• Depression
					• Anxiety
*Lessertia frutecens*	Cancer bush	Southern Africa	Southern Africa	Decoctions from leaves or bark	• Cancer
					• Viral infections; specifically, HIV/AIDS
*Moringa oleifera*	Moringa	India	Widespread	Leaf powder: other parts also used	• Diabetes
					• Inflammation
*Uncaria tomentosa*	Cat’s claw	South/Central America	Widespread	Bark	• Viral infection
					• Inflammation
*Vernonia amygdalina*	Bitter leaf	Southern Africa	Southern Africa	Leaf infusion	• Diarrhoea
					• Constipation
					• Stomachache
					• Malaria
					• Diabetes
					• Cancer
					• Pyrexia

### Andrographis (*Andrographis paniculata*)

*Andrographis paniculata,* also known as green chiretta, belongs to the Acanthaceae family, has Ayurvedic origins, and is native to India and Sri Lanka. During the COVID-19 pandemic, China, Thailand, and other countries incorporated this herbal medicine into treatment guidelines and added it to essential medicine lists (Intharuksa et al., 2022). *A. paniculata* leaf extract has been used to treat ailments such as stomachache, inflammation, pyrexia, loss of appetite, irregular stools, and diarrhoea. The aerial parts have been documented in the treatment of the common cold, hypertension, malaria, and snakebites (Okhuarobo et al., 2014). *A. paniculata* has the potential for HDIs, particularly when taken in combination with drugs metabolized by CYP3A, CYP2C9, and CYP2C19, through both induction and inhibition (Sundhani et al., 2022). Balap et al. (2017) reported that co-administration of *A. paniculata* extract with naproxen, a CYP2C9 substrate in humans, decreased systemic exposure to naproxen in rat models. Systemic concentrations of drugs metabolized by CYP1A2 increased after co-administration with *A. paniculata* in rat models (Chien et al., 2010). With the increased use of this herbal medicine during the COVID-19 pandemic, HDIs with Paxlovid™ through CYP inhibition and induction, are possible.

### Sweet wormwood (*Artemisia annua*)

*Artemisia annua* L., also known as sweet wormwood, is a member of the Asteraceae family and has a long history as treatment for various diseases. Extracts and isolated phytochemicals of *A. annua* have been reported to have anti-viral, pro-apoptotic, anti-inflammatory, anti-pyretic, antioxidant, and immunomodulatory activities (Soni et al., 2022). The sesquiterpenes produced by *A. annua*, artemisinin and artesunate, have shown high efficacy against multi-drug resistant malaria-causing parasites, resulting in artemisinin-based combination therapies for the treatment of *P. falciparum* malaria (Bosman et al., 2007). Artemisinin is known to be both an inducer and a substrate of CYP3A4 and CYP2B6 (Xing et al., 2012), leading to concerns about herb-drug interactions with artemisinin-based combination therapy. Based on its relatively broad range of biological activities, *A. annua* has also been used for the treatment of rheumatoid arthritis, systemic lupus erythematosus, allergic contact dermatitis, and has been evaluated for treatment of COVID-19 (Fuzimoto, 2021; Soni et al., 2022). *A. annua* extracts demonstrated inhibition of CYP3A4 activity (measured via 6ẞ-(OH)-testosterone) in Caco-2 cell monolayers, whereas artemisinin alone had no effect (Melillo de Magalhães et al., 2012). The same study also showed inhibition as well as a slight induction of CYP1A1 activity (measured via 7-ethoxyresorufin-O-deethylase) in Caco-2 cell monolayers (Melillo de Magalhães et al., 2012). *A. annua* tea infusions showed inhibition of CYP2B6 and CYP3A4 activity in HepaRG cells using P450-Glo assays (Kane et al., 2022). Based on the data available, HDIs with Paxlovid™ through CYP inhibition and induction with *A. annua* are possible.

### Climbing onion (*Bowiea volubilis*)

*Bowiea volubilis* Harv. ex Hook.f. is a perennial, succulent plant commonly known as climbing onion, that belongs to the family Asparagaceae and is widely grown in dry regions of eastern and southern Africa (Ramarumo et al., 2019). Among the tribes of Southern Africa, the bulbous medicinal plant is traditionally used to treat various medical conditions, including dermatological disorders, sore eyes, urinary complications, infertility, and facilitation and induction of abortion (Steenkamp, 2003). Most preparations for therapeutic use involve mixing crushed fresh bulbs with water to form a decoction. A study by Fasinu et al. (2014) (Fasinu et al., 2014) showed that aqueous extracts of *Bowiea volubilis* inhibited the activity of CYP1A2 (phenacetin O-deethylation) and CYP3A4 (testosterone 6β-hydroxylation) in human liver microsomes, suggesting the potential for HDIs if these concentrations are attained *in vivo*. Studies have reported that all parts of *B. volubilis* contain alkaloids and several cardiac glycosides of the scillaren type (Mulholland et al., 2013). The mechanism of action of cardiac glycosides involves binding and inhibition of Na^+^/K^+^-ATPase with high selectivity and affinity. The CYP inhibitory activity of *B. volubilis* may be attributed to the alkaloids, which are substrates of human CYPs. A study by Salminen et al. (2011) showed that structurally related alkaloids from plants inhibited major human drug metabolizing CYPs (CYP3A4, CYP2D6, CYP2C19). Further research is needed to determine whether *B. volubilis* precipitates interactions with Paxlovid™ in patients and potential clinical consequences.

### Turmeric (*Curcuma longa*)

Turmeric is obtained from the rhizomes of *C. longa* L., a plant of the Zingiberaceae family that is native to tropical South Asia. The rhizomes are yellow-brown and are usually dried and ground to a yellow powder for use as a spice, herbal medicine, or dietary supplement. Ayurvedic medicine, Chinese medicine, and various pharmacopoeias describe uses of turmeric that include treatment of peptic ulcers, allergy, pain and inflammation, and liver disorders (Razavi et al., 2021). Turmeric is also under investigation for potential anti-SARS-CoV-2 activity and possible value in mitigating the COVID-19 cytokine storm (Rattis et al., 2021). The rhizomes of *Curcuma longa* contain monoterpenes and sesquiterpenes, including turmerone, arturmerone, and zingiberene, polysaccharides and curcuminoids. Turmeric dietary supplements usually contain extracts enriched in curcuminoids (up to 95% by weight) (Cheng et al., 2019). Some preclinical and clinical studies suggest that turmeric may produce pharmacokinetic HDIs. In a prospective study of 60 breast cancer patients, co-administration of turmeric with paclitaxel reduced serum AUC and C_max_ of paclitaxel by 7.7% and 12.1%, respectively (Kalluru et al., 2022). Although probably caused by induction of CYP3A4 activity, the effect was not considered clinically relevant. A study involving a human colorectal adenocarcinoma cell line showed curcumin inhibits P-gp, indicating the potential to alter the pharmacokinetics of drugs that are substrates for this efflux transporter (Flory et al., 2021). A clinical study involving eight healthy adult participants showed that curcumin increased the area under the curve (AUC) of sulfasalazine, a substrate for BCRP (Kusuhara et al., 2012). Several uridine 5′-diphospho-glucuronosyltransferase (UDP-glucuronosyltransferase, UGTs), including hepatic UGT1A1 and intestinal UGT1A8 and UGT1A10, metabolize curcumin to curcumin-*O-*glucuronide, which is a substrate for the basolaterally located efflux transporter MRP3 (ABCC3) (Jia et al., 2020). Therefore, pharmacokinetic turmeric-drug interactions may occur for drugs that are also substrates for MRP3 or these UGTs.

### Licorice (*Glycyrrhizae radix et rhizoma*)

*Glycyrrhizae radix et rhizoma* (Chinese name, Gan cao), also known as licorice, is the dried root and rhizome of *Glycyrrhiza uralensis* Fisch., *Glycyrrhizae inflata* Bat, or *Glycyrrhizae glabra* L, and is a commonly used herbal medicine native to West Asia, North Africa, and Southern Europe (Wang et al., 2013; Liu et al., 2021). Licorice is used as an herbal medicine, dietary supplement, and food flavouring (Omar et al., 2012), and has been reported to exhibit various bioactivities, including anti-allergy, anti-inflammation, anti-virus, and anti-oxidant effects (Zhao et al., 2017). Studies have shown that licorice extracts or its bioactive phytochemicals can inhibit SARS-CoV-2 infection by affecting entry and replication of the virus, having potential for the treatment of COVID-19 (Sprouse and van Breemen, 2016; Yi et al., 2022). Various natural products and Chinese herbal formulae against COVID-19 are recommended by the Guidelines of Diagnosis and Treatment for COVID-19 issued by the National Health Commission of China. Licorice is one of the most frequently involved herbs in these herbal formulae or preparations against COVID-19, increasing risk for HDIs for anti-COVID-19 drugs (Lyu et al., 2021), and has been reported to modulate several CYP isoforms *in vitro*, including CYP3A4, CYP2C9, and CYP2E1 (Wang et al., 2013). Licorice extract was shown to activate human pregnane X receptor (PXR) (Haron et al., 2022) and induce CYP3A4 activity in human hepatoma cells (Wang et al., 2012). Regarding drug transporters, glycyrrhizin, one of the main bioactive ingredients of licorice, was shown to inhibit MRP2's uptake of S-(2,4-dinitrophenyl)-glutathione and induce P-gp. (Feng et al., 2015). Conversely, ritonavir is an inducer of uridine diphosphate (UDP)-glucuronyltransferase (UGT) and an inhibitor of P-gp and breast cancer resistance protein (BCRP), which may decrease efflux and in turn increase the metabolism of licorice phytochemicals (Marzolini et al., 2022). Given that licorice is one of the most used herbal medicines, in addition to a promoted treatment against COVID-19, there is potential concern regarding interactions with Paxlovid™.

### Devil’s claw (*Harpagophytum procumbens*)

*Harpagophytum procumbens* is a weedy, perennial, and herbaceous plant that is geographically distributed in the Kalahari Desert of Southern Africa. (Mncwangi et al., 2012). Harpogoside was isolated as the major phytochemical from *H. procumbens* tubers, amongst other iridoid glycosides. Both the pure isolated phytochemical (harpagoside) and extracts from the plant have shown potent anti-rheumatic, anti-inflammatory and analgesic effects (Georgiev et al., 2013). Products containing devil’s claw extract are widely used internationally by patients suffering from chronic low back pain and/or other chronic inflammatory diseases (Grant et al., 2007). Unger and Frank (2004) (Unger and Frank, 2004) conducted a study using baculovirus-infected insect cells to measure inhibition of the activity of six major CYPs by an *H. procumbens* extract, amongst other herbal extracts using LC/LC/MS. Inhibition of CYP1A2 and CYP2D6 by *H. procumbens* extract was comparably low, whereas CYP2C8, CYP2C9, CYP2C19 and CYP3A4 showed higher levels of inhibition. In another study, the effects of three commercially available devil’s claw products on P-gp activity were tested in a human proximal tubule (HK-2) cell line. (Romiti et al., 2009). Two of the products inhibited P-gp activity in calcein-AM tests and decreased protein expression via Western blot, albeit to a lower extent than the positive control, verapamil. These commercial devil’s claw products also inhibited esterase activity (via free calcein measurements), which may have resulted in an underestimation of their true P-gp inhibitory effects. However, harpagoside alone did not inhibit P-gp activity nor esterase activity (Romiti et al., 2009). Due to these *in vitro* interactions observed for devil’s claw through CYP and P-gp inhibition, Bordes et al. (2020) (Bordes et al., 2020) noted that *H. procumbens* may increase plasma concentrations of anti-retroviral drugs in patients, including those containing ritonavir, when administered concomitantly.

### African potato (*Hypoxis haemercollidea*)

*Hypoxis haemercollidea* Fisch., C.A. Mey. (Hypoxidaceae), also known as African potato, is a tuberous perennial with star shaped yellow flowers that is indigenous to Southern Africa (South Africa, Lesotho, Eswatini, Zimbabwe, Botswana, and Mozambique) (Matyanga et al., 2020). Many species of *Hypoxis* are used as herbal medicines and are often morphologically similar (Ncube et al., 2013). Of these, *H. haermercollidea* is the best known and most studied. Decoction of *H. haermercollidea* corms is the most prominent herbal medicine preparation specifically used to treat HIV/AIDS as an immunomodulator (Mills et al., 2005a) and is also used to treat tuberculosis, cancer, headache, dizziness, ulcers, seizure disorders, depression, and anxiety (Ncube et al., 2013). Hypoxoside is the main phytochemical in African potato, which is readily converted to rooperol by β-glucosidase. Rooperol is the biologically active phytochemical that is attributed to the medicinal properties of the plant (Drewes and Khan, 2004; Mills et al., 2005a). Other notable biologically active phytochemicals are phytosterols and their glycosides, specifically β-sitosterol (Mills et al., 2005a; Nair et al., 2007; Matyanga et al., 2020). HDIs involving *H. haemercollidea* have been investigated, most focusing on antiretroviral drugs. African potato extracts mainly inhibited CYP3A4 (Mills et al., 2005b; Nair et al., 2007; Gwaza et al., 2009), as well as CYP1A2, 2A6, 2B6, 2C9, 3A5, and 2D6 in vitro assays (Nair et al., 2007; Gwaza et al., 2009; Fasinu et al., 2013). African potato extract showed dose-dependent activation of PXR (Mills et al., 2005b). For extracts high in hypoxoside, P-gp-mediated efflux increased compared to the ritonavir control (Nair et al., 2006). The P-gp-mediated efflux of nevirapine across human intestinal epithelial cells increased significantly in the presence of an African potato extract compared to vehicle (Brown et al., 2008), whereas the P-gp-mediated efflux of indinavir decreased substantially in the presence of *H. haemercollidea* (Havenga et al., 2018) compared to controls. Other studies showed no induction of P-gp at the concentrations tested (Gwaza et al., 2009; Fasinu et al., 2013). Co-administration of African potato with ART in human clinical trials did not alter the pharmacokinetics of efavirenz (Mogatle et al., 2008) nor lopinavir/ritonavir (Gwaza et al., 2013), which are substrates for and inhibitors of CYP2B6 and CYP3A4, respectively. Although the Gwaza et al. (2013) study showed no change in drug pharmacokinetics with concomitant use of *Hypoxis obtusa*, additional studies are needed to further elucidate potential concern with use of *H. haemercollidea* extracts co-administered with Paxlovid™, particularly regarding potential P-gp-mediated interactions.

### Cancer bush (*Lessertia frutecens*)

*Lessertia frutecens* (L.) Goldblatt & J.C.Manning (previously *Sutherlandia frutecens* (L.)., R.Br) also known as cancer bush or Sutherlandia, is a flowering shrub that is a member of the Fabaceae family (Mills et al., 2005a; van Wyk and Albrecht, 2008). The plant is indigenous to South Africa, Lesotho, southern Namibia, and south-eastern Botswana and has been used by a wide range of cultures as a medicinal plant (van Wyk and Albrecht, 2008). Cancer bush has been used mainly to treat internal cancers—hence the name—as well as diabetes, inflammation, and infections, and to promote wound healing (van Wyk and Albrecht, 2008). More recently, *Lessertia frutecens* has gained popularity as antiviral treatment, specifically against HIV/AIDS as an immunomodulator (Mills et al., 2005a; Babb et al., 2007; Sibanda et al., 2016). Traditionally, decoctions or infusions are made from the leaves or bark (van Wyk and Albrecht, 2008). The main phytochemicals of *L. frutescens* are L-canavanine, D-pinitol, and gamma-aminobutyric acid (Mills et al., 2005a; van Wyk and Albrecht, 2008). Although research is limited, *Lessertia* has shown the potential to precipitate HDIs, specifically with antiretroviral drugs. Both water and ethanolic extracts showed inhibition of CYP3A4 (via conversion of dibenzylfluorescein to fluorescein) and activation of hPXR (via CYP3A4 luciferase reporter gene construct) and inhibited P-gp activity (via orthovanadate sensitive release of phosphate and adenosine triphosphatase activity) in HepG2 cells (Mills et al., 2005b). Similarly, *Lessertia* extracts inhibited P-gp in MDCK-MDR1 cells, reducing amprenavir efflux (Katerere, 2018), and modestly inhibited P-gp in Caco-2 cells using nevirapine as the substrate (Brown et al., 2008). In the same study by Brown et al. (2008), inhibition of P-gp by L-canavanine, a main phytochemical of *L. frutescens*, was observed. Extracts of *L. frutecens* inhibited a range of CYPs but mainly CYP3A4 and CYP3A5 (Fasinu et al., 2013). In LS-180 cells, CYP3A4 decreased after a short-term exposure to a *Lessertia* aqueous extract (less than 5 days); however, after 5 days of exposure, CYP3A4 activity increased 2-3 fold (Minocha et al., 2011). The lack of increase in CYP3A4 mRNA expression implied a post-transcriptional mechanism. In the same study, the pharmacokinetics of nevirapine were altered in rats. Specifically, the area under the curve (AUC) and maximum plasma concentration (C_max_) decreased by 50% after 5 days of treatment with *Lessertia* (Minocha et al., 2011). Similarly, the AUC and C_max_ of atazanavir decreased in human participants (n = 12) after a single dose of Sutherlandia tablets (Müller et al., 2013). The activation of CYP3A4 and inhibition of P-gp by *L. frutecens* has the potential to elicit sub-therapeutic effects of the drugs administered, which may lead to either treatment failure or drug resistance. Given these potential interactions with CYPs and related pathways, there is evidence for potential HDIs with *Lessertia.*

### Moringa *(Moringa oleifera)*

*Moringa oleifera*, commonly known as moringa, is a tree that is native to India and widely cultivated throughout the tropics and subtropics because of its adaptability to different climatic conditions. (Kashyap et al., 2022; Liu et al., 2022). Previously reported analgesic, anti-inflammatory, antipyretic and immune boosting properties render moringa a potentially viable treatment for the management of COVID-19 (Chikowe et al., 2021; Nuertey et al., 2022; Siddiqui et al., 2022). However, some preclinical and clinical safety data indicate the potential for HDIs. Inhibition of CYP1A2 and CYP3A activity in pooled human liver microsomes has been reported. Using testosterone as the probe substrate, methanolic leaf extracts of *M. oleifera* inhibited CYP3A activity modestly, suggesting low interaction risk. Likewise, aqueous extracts showed weak CYP3A inhibition (Monera et al., 2008). Using fluorometric probes, methanolic extracts of *M. oleifera* leaves inhibited CYP3A and CYP1A2 activity (Awortwe et al., 2014), and aqueous extracts of *M. oleifera* leaves inhibited CYP1A2 and CYP2C9 activity. Inhibition of CYP3A and CYP1A2 activity was considered most likely because the IC_50_s were higher than the minimum estimated dose warranting further investigation (Awortwe et al., 2014; Showande et al., 2019). Additionally, inhibitory effects of an ethanolic extract of moringa leaves on CYP1A2 and CYP3A4 activity were demonstrated *in vitro* (Taesotikul et al., 2010; Ahmmed et al., 2015). Two human clinical trials assessing the interaction between moringa and pharmaceutical drugs compared AUC and C_max_ of nevirapine with and without moringa coadministration, showing that moringa had no effect on the steady-state pharmacokinetics of the drug based on the FDA bioequivalence approach (USFDA, 2016; Monera-Penduka et al., 2017). In contrast, coadministration of moringa with amodiaquine resulted in a significant decrease (*p* = 0.037) in the time for amodiaquine to reach C_max_. Several moringa phytochemicals have demonstrated antispasmodic activity, including glucomorigin, which may slow absorption, thus increasing time to reach C_max_. In addition, other phytochemicals could form insoluble complexes affecting both C_max_ and the time to reach C_max_ (Olawoye et al., 2018). Although additional data are needed, some preclinical and clinical safety data indicate the potential for interactions between moringa and Paxlovid™.

### Cat’s claw (*Uncaria tomentosa*)

Cat’s claw (*Uncaria tomentosa*) has shown beneficial effects against inflammation and viral infection, including COVID-19 (Steinberg, 1995; Erowele and Kalejaiye, 2009; Yuan et al., 2014; Yepes-Perez et al., 2021). Multiple *in vitro* studies have demonstrated cat’s claw as a CYP3A inhibitor (Budzinski et al., 2000; Sato et al., 2015; Weiss, 2018), but the specific phytochemicals have not been identified. A clinical study showed cat’s claw to inhibit CYP3A, as evidenced by increased plasma concentrations of antiretroviral drugs that are CYP3A substrates, including ritonavir (López Galera et al., 2008). In addition, a cell-based study showed that an extract of cat’s claw upregulated the protein expression of CYP2J2, CYP3A4, UGT1A3, UGT1A9, P-gp, and the apically located uptake transporter organic anion transporting polypeptide (OATP) 1B1 (SLCO1B1) (Weiss, 2018). Both nirmatrelvir and ritonavir are CYP3A substrates (Kumar et al., 1996; Li et al., 2011; Eng et al., 2022), suggesting risk for HDIs between cat’s claw and Paxlovid™ via CYP3A inhibition or induction.

### Bitter leaf (*Vernonia amygdalina*)

*Vernonia amygdalina* is a small, perennial, rapidly regenerating shrub that grows wild in many sub-Saharan African countries, particularly Nigeria, Cameroon, South Africa, and Zimbabwe. Commonly known as bitter leaf, *Vernonia amygdalina* is widely consumed as a green leafy vegetable. An infusion of *V. amygdalina* leaves, sometimes with the young stems, is used to treat gastrointestinal tract conditions such as diarrhoea, constipation, and stomachache caused by helminthic, protozoal, and bacterial infection. *Vernonia amygdalina* is also used to manage malaria, diabetes, and cancer (Toyang and Verpoorte, 2013; Oyeyemi et al., 2018; Gyebi et al., 2021). Its use in managing fever and colds made it a candidate for screening as a potential source for a COVID-19 drug target (Gyebi et al., 2021). *Vernonia amygdalina* contains saponins, alkaloids, sesquiterpenes, and steroid glycosides, which are responsible for the plant’s biological activity (Farombi and Owoeye, 2011). *Vernonia amygdalina* has been shown to inhibit P-gp activity *in vitro* and to increase systemic exposure to the P-gp probe drug digoxin *in vivo*. Using Caco-2 cells, aqueous extracts of *V. amygdalina* leaves significantly inhibited P-gp-mediated efflux digoxin (*p* < 0.01) relative to control (Farombi and Owoeye, 2011; Oga et al., 2012). Using an isolated rat ileum model, *V. amygdalina* increased digoxin permeability in the mucosal-to-serosal direction by 43% compared to control (Oga et al., 2013). When *V. amygdalina* was co-administered with digoxin to rats, total plasma digoxin AUC was 2.1 times higher than that after digoxin was administered with vehicle. Caution may be warranted when *V. amygdalina* is co-consumed with other P-gp substrate drugs’ however, further research is needed to determine whether these pre-clinical results translate to the clinical setting.

## Discussion/conclusion

Herbal medicines are used around the world in the treatment and prevention of disease. The rollout of Paxlovid™ during the COVID-19 pandemic has prevented hospitalizations and saved lives and will continue to as it becomes more globally available (Shah et al., 2022; Wong et al., 2022). Comprehensive resources have been prepared to help address challenges that the use of Paxlovid™ presents. For example, the United States Food and Drug Administration (FDA) created an interaction checklist and the University of Liverpool designed a COVID-19 drug interaction tool (USFDA, 2022c; Liverpool, 2022). However, these resources do not account for the impact of HDIs on patient care beyond St. John’s wort.

The herbal medicines highlighted above, although not comprehensive, demonstrate the potential for interactions with ritonavir-containing medications, including Paxlovid™, particularly through CYP induction or inhibition. Because herbal medicine use varies across regions, public health bodies and ministries of health may consider creating an interaction checklist (similar to FDA’s or Liverpool’s checklist (USFDA, 2022c; Liverpool, 2022)) and include commonly used herbal medicines that have potential HDIs with Paxlovid™ alongside drug-drug interactions. These checklists can be accompanied with education for both the public and healthcare providers to raise awareness of potential HDIs by encouraging patients to report herbal medicine use to their providers and by equipping providers with tools and knowledge to manage such HDIs.

Extensive *in vitro* and *in silico* data are available for HDIs involving herbal medicines such as *V. amygdalina*, and *L. frutecens*. However, how these data and results translate to the clinical settings remain unknown. As Paxlovid™ is scaled up globally and the concurrent use of antivirals and herbal medicines increases, additional research can help elucidate the clinical impact of HDIs. Prospective and retrospective studies can add to the understanding of HDIs. Predictive modelling based on available non-clinical data by applying established tools (e.g., physiology-based pharmacokinetic modelling) may also inform HDIs with Paxlovid. Without this critical information, adverse events can result, and systemic concentrations can vary (both for Paxlovid™ and herbal medicine phytochemicals), which may lead to drug resistance, low patient adherence, and failed therapy.

Study limitations include a non-systematic review methodology, however, the information provided in this manuscript raises awareness of potential HDIs and is not meant to be a comprehensive review.

With the likelihood of future SARS-CoV-2 variants emerging, Paxlovid™ represents an effective therapeutic tool to prevent death and strain on healthcare systems. Raising awareness and furthering the understanding of HDIs is crucial as the global Paxlovid™ rollout continues.

## Footnotes

1 See, e.g., 45 C.F.R. part 46, 21 C.F.R. part 56; 42 U.S.C. §241(d); 5 U.S.C. §552a; 44 U.S.C. §3501 et seq.
